# Serine protease inhibitors and human wellbeing interplay: new insights for old friends

**DOI:** 10.7717/peerj.7224

**Published:** 2019-08-30

**Authors:** Héla Mkaouar, Nizar Akermi, Aicha Kriaa, Anne-Laure Abraham, Amin Jablaoui, Souha Soussou, Raja Mokdad-Gargouri, Emmanuelle Maguin, Moez Rhimi

**Affiliations:** 1 INRA, UMR1319 MICALIS, Jouy-en-Josas, France, AgroParisTech, UMR MICALIS, Jouy-en-Josas, France; 2 MaIAGE, INRA, Université Paris-Saclay, Jouy-en-Josas, France; 3 Laboratory of Molecular Biology of Eukaryotes, Center of Biotechnology of Sfax, University of Sfax, Sfax, Tunisia

**Keywords:** Serine protease inhibitors, Disease, Homeostasis, Function, Human gut microbiota

## Abstract

Serine Protease Inhibitors (Serpins) control tightly regulated physiological processes and their dysfunction is associated to various diseases. Thus, increasing interest is given to these proteins as new therapeutic targets. Several studies provided functional and structural data about human serpins. By comparison, only little knowledge regarding bacterial serpins exists. Through the emergence of metagenomic studies, many bacterial serpins were identified from numerous ecological niches including the human gut microbiota. The origin, distribution and function of these proteins remain to be established. In this report, we shed light on the key role of human and bacterial serpins in health and disease. Moreover, we analyze their function, phylogeny and ecological distribution. This review highlights the potential use of bacterial serpins to set out new therapeutic approaches.

## Introduction

Serpins were first discovered in 1980 when Hunt and Dayhoff noticed similarities between ovalbumin, an egg white protein and two human proteins: antithrombin and *α*1-antitrypsin (*α*1-AT) (Hunt & Dayhoff, 1980). The acronym serpin was coined in 1985 to designate serine protease inhibitors (Carrell & Travis, 1985). Serpins constitute a superfamily displaying different functions and are divided into 16 clades (named A-P) (Heit et al., 2013). Although serpin acronym initially derived from their main function, which is the inhibition of serine proteases (Gettins, 2002; Huntington, 2011), cross-class inhibition was also demonstrated (Schick et al., 1998; Bao et al., 2018). However, several serpins do not exhibit any inhibitory activity but coordinate a wide range of other biological functions (Hammond et al., 1987; Clarke et al., 1991; Gettins, 2002; Carrell & Read, 2017). In human, serpins are well studied and their dysregulation is often associated to many pathologies including inflammation, cardiovascular diseases, cancer and neurological disorders (Ho et al., 1994; Wolf et al., 1999; Vecchi et al., 2008). Many reports stressed the key role of serpins in human health leading to their suggestion as potential therapeutic targets (Richardson, Viswanathan & Lucas, 2006; Zheng et al., 2013; Al-Horani, 2014).

Unlike eukaryotic serpins, the discovery of their prokaryotic counterparts is relatively recent. Indeed, until 2002, serpins were believed to be restricted to eukaryotes, but based on phylogenetic analysis, Irving et al. (2002) evidenced that such proteins are also encoded by prokaryotes (Irving et al., 2002). Despite these findings, bacterial serpins remain poorly studied and data about their origin and functions need to be established.

In this review, we report a concise overview of serpin functions in human and outline the current knowledge on bacterial serpins. Moreover, we provide the first analysis of serpins encoded by human gut microbiota and their impact on host wellbeing.

### Survey methodology

In this review, we discussed the current literature related to serpins and their functions in health and disease, with a focus on the human gut microbiota. References mentioned in this review were retrieved from PubMed up to 2019. We used the research terms such as serpin, microbiota, health and diseases. Considered references will provide more information about serpins and their impact on the human health. We excluded the studies related to the serpin engineering and the improvement of their biochemical behaviors. Protein sequences encoding for serpins were isolated from the NCBI public database using the key word “serpin”. Phylogenetic tree was built with PhyloT (https://phylot.biobyte.de/) and ITOL.

### Human serpins

Serpins were extensively studied in eukaryotes. Since 2012, the number of genes encoding eukaryotic serpins listed in NCBI has increased from 6,628 to 12,953 (Gaci et al., 2013). Human genome encodes 37 serpins, among them 30 are functional inhibitors (Rau et al., 2007; Lucas et al., 2018; Sanrattana, Maas & De Maat, 2019). They act at various cellular compartments and they are involved in many physiological functions. In fact, these inhibitors are encoded by 10 different chromosomes and belong to the A-I clades (Heit et al., 2013). Most serpins from clade A i.e., extracellular serpins, are encoded by a group of genes located on chromosome 14 and act through the regulation of protease activities involved mainly in: pathogen invasion, injury and inflammation (Olson & Gettins, 2011). While in clade B, serpins (known as ov-serpins) are intracellular and are encoded by genes from chromosomes 6 and 18 (Gettins, 2002; Olson & Gettins, 2011). Given their mechanism of inhibition, serpins were selected to control tightly regulated physiological processes (Huntington, 2011) such as the blood coagulation cascade (Anti-thrombin) (Pickering & Hewitt, 1922; Quinsey et al., 2004; Pike et al., 2005; Hepner & Karlaftis, 2013) and tissue remodeling (Plasminogen Activator Inhibitor-1 and 2) (Diebold et al., 2008). Serpins also play key roles in other processes including the control of the inflammatory response (Anti-trypsin, Anti-chymotrypsin) (Bots & Medema, 2008), programmed cell death and cell development (Bird, 1998; Kryvalap et al., 2018). Moreover, Serpins display functions such as blood pressure regulation (SERPINA8) (Davisson et al., 1997), hormone transport (SERPINA6, SERPINA7) (Zhou et al., 2006; Klieber et al., 2007), tumor suppression (SERPINB5) (Zhang, Magit & Sager, 1997) as well as molecular chaperone functions (SERPINH1) (Silverman et al., 2010) which are not based on protease inhibition.

In agreement with their functions, serpin disequilibrium is associated to several physiopathologies in humans (Table 1). The expression of *α*1-AT is altered in patients suffering from inflammatory bowel diseases (IBD) (Karbach, Ewe & Bodenstein, 1983; Grill, Hillemeier & Gryboski, 1984). Hence, the administration of this protein attenuated the intestinal inflammation in mice by reducing the cellular infiltration and the secretion of pro-inflammatory cytokines as well as restoring the epithelial barrier and limiting tissue damage (Collins et al., 2013). Moreover, it was described that SERPINE1 was associated to lung inflammation (Table 1). Serpins are also involved in obesity as demonstrated for vaspin (visceral adipose tissue-derived serpin). Clinical data revealed an increase of vaspin level in adipose tissues from obese and type 2 diabetes patients (Cho, Han & Kang, 2010; Klöting et al., 2011; Zhang et al., 2011; Teshigawara et al., 2012). Furthermore, the administration of vaspin to obese mice improved glucose tolerance and insulin sensitivity (Hida et al., 2005). Such beneficial effect was linked to the inhibition of KLK7 (Kallikrein-Related Peptidase 7) which is up-regulated in obesity-induced insulin resistance patients (Hida et al., 2005; Heiker et al., 2013). In addition to that, it was suggested that blocking serpinB13 might prevent the development of type1diabetes (Table 1). Serpins are also believed to be involved in cardiovascular diseases. In fact, Kallistatin, a protease inhibitor widely distributed in tissues relevant to cardiovascular function (Chai et al., 1993; Chao & Chao, 1995; Chao et al., 1996; Wolf et al., 1999), is significantly reduced in coronary artery disease (Chao, Guo & Chao, 2018). This protein displays many properties including anti-atherosclerotic effects and reduction of infarct size (Chao et al., 2006; Gao et al., 2008; Shen et al., 2010). Besides metabolic and inflammatory disorders, many studies reported the clinical relevance of serpins in cancer. In this context, it was reported that Maspin, a non-inhibitory serpin, is significantly associated to breast and prostate cancers (Cao et al., 2007; Vecchi et al., 2008). Increased level of Maspin was detected in different types of cancer and shown to (i) efficiently promote cancer cell apoptosis, (ii) exhibit anti-angiogenesis activity and (iii) inhibit cancer cell migration (Zou et al., 1994; Zhang et al., 1999; Ngamkitidechakul et al., 2001; Song et al., 2002; Cher et al., 2003; Sopel, Kasprzyk & Berdowska, 2005). In contrast, it was recently demonstrated that Maspin cannot be considered as a tumor suppressor but may be a prognostic indicator (Teoh et al., 2014). In addition to Maspin, SERPINE2 and SERPINF1 are associated to many carcinoma types including lung, prostate, pancreatic and papillary thyroid cancers (Halin et al., 2004; Zhang et al., 2006; Stepień et al., 2017). Based on these findings, serpins appear as attractive therapeutic targets to set out new medical strategies against some human pathologies.

**Table 1:** Biological functions of serpins and their association to human diseases.

**Clade**	**Serpin**	**Biological function**	**Associated disease**	**Reference**
A	*α*1-Antitrypsin (SERPINA1)	Complement activation, apoptosis	Emphysema, Cirrohosis, IBD, Cancer (liver)	Eriksson, Carlson & Velez (1986), Lomas et al. (1992), Yang et al. (2000), Saunders et al. (2012) and Heit et al. (2013)
	Antichymotrypsin (SERPINA3)	Complement activation, apoptosis, prohormone conversion	Emphysema, Alzheimer’s disease	Law et al. (2006), Kamboh et al. (2006) and Heit et al. (2013)
	Kallistatin (SERPINA4)	Complement activation, angiogenesis, fibrinolysis, apoptosis	Coronary artery, Hypertension, Cardiovascular diseases, Chronic liver diseases	Chao et al. (1996), Heit et al. (2013) and Nallagangula et al. (2017)
	Coticosteroid -binding globulin (SERPINA6)	Hormone transport	Chronic fatigue	Torpy et al. (2004)
	Thyroxine-binding globulin (SERPINA7)	Hormone transport	Hypothyroidism	Refetoff et al. (1996) and Moeller et al. (2015)
	Angiotensinogen (SERPINA8)	Blood pressure regulation, hormone transport	Hypertension	Kim et al. (1995) and Yan et al. (2018)
	Protein Z-dependent proteinase inhibitor (SERPINA10)	Inhibition of factor Z and XI	Venous thromboembolic disease	Van de Water et al. (2004) and Law et al. (2006)
	Vaspin (SERPINA12)	Insulin-sensitizing adipocytokine	Obesity, Insulin resistance, Diabetes	Hida et al. (2005) and Heiker (2014)
B	Plasminogen activator inhibitor-II (SERPINB2)	Fibrinolysis, elastase inhibitor	Cancer	Medcalf & Stasinopoulos (2005), Su et al. (2015) and Harris et al. (2017)
	Squamous cell carcinoma antigen-I/II (SERPINB3/B4)	Anti-apoptosis	Respiratory and skin inflammatory diseases	Sun, Sheshadri & Zong (2017) and Izuhara et al. (2018)
	Maspin (SERPINB5)	Anti-angiogenesis	Cancer (breast, prostate, colon, bladder)	Zou et al. (1994) and Berardi et al. (2013)
	Megsin (SERPINB7)	Renal development, Mesangial cell proliferation	IgA nephropathy	Miyata et al. (1998)
C	Antithrombin (SERPINC1)	Coagulation, angiogenesis	Thrombosis, Lung inflammation	Perry & Carrell (1996) and Ishikawa et al. (2017)
D	Heparin cofactor II (SERPIND1)	Coagulation	Thrombosis, Cancer (lung)	He et al. (2002) and Liao et al. (2015)
E	Plasminogen activator inhibitor I (SERPINE1)	Angiogenesis, fibrinolysis, anti-apoptosis	Bleeding disorders, Cancer, Septic shock, acute lung inflammation	Law et al. (2006), Placencio et al. (2015), Gupta et al. (2016) and Ozolina et al. (2016)
F	PEDF (SERPINF1)	Anti-angiogenesis	Cancer (prostate, melanoma)	Becerra & Notario (2013)
	Alpha-2-antiplasmin (SERPINF2)	Fibrinolysis	Bleeding disorders	Miles et al. (1982)
G	C1 inhibitor (SERPING1)	C1 esterase inhibitor	Angioedema	De Marchi et al. (1973)
H	Heat shock protein (SERPINH1)	Chaperone	Osteogenesis imperfecta	Nagata (1996) and Lindert et al. (2015)
I	Neuroserpin (PII4) (SERPINI1)	Neutrofic factor	Dementia	Davis et al. (1999)

### Serpins structure

**Figure 1:**
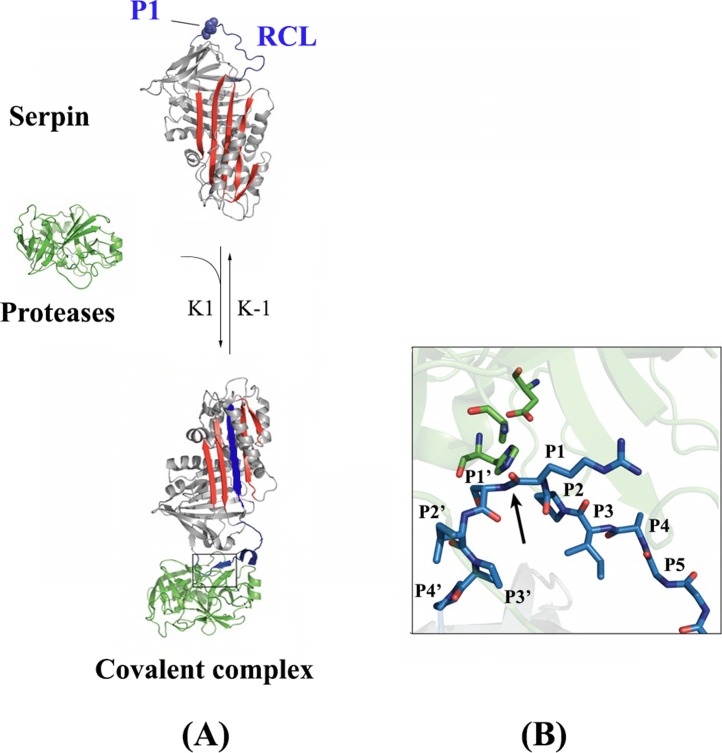
Serpin structure and mechanism of inhibition. (A) The RCL (Blue) is recognized by a serine protease (green). After cleavage, RCL rapidly inserts into *β*-sheet and forms a covalent serpin-protease complex. (B) Close-up view of the interaction between the serpin and its target protease (adapted from Song et al., 2011, permission license number 4545950475192).

Many structural and biochemical analysis provided a major knowledge progress on serpin family. Serpins display a single domain of 40–60 kDa (PFAM ID PF00079) with an average size of 350–400 amino acids (Stein & Carrell, 1995; Irving et al., 2000; Gettins, 2002). Currently, around 200 three-dimensional structures of serpin and serpin-protease complexes are available in PDB database deriving from both eukaryotes and prokaryotes that display significant structural similarities. Most of these structures (∼90%) belong to eukaryotic species, while only three serpins structure from thermophilic and pathogenic bacteria are solved (Irving et al., 2003; Fulton et al., 2005; Zhang et al., 2007; Goulas et al., 2017). Overall, serpins shared a common fold in spite of their low sequences homology (∼25%) (Huntington, 2011). Serpin architecture is typically composed of 3 *β*-sheets (A, B and C), 8-9 *α*-helices (named hA–hI) and a Reactive Center Loop (RCL) (Fig. 1A). The latter is a long and flexible loop (20–25 amino acids linking the *β*-sheets A and C) that mediates the conformational conversion during the protease docking and inhibition (Gettins, 2002; Law et al., 2006; Huntington, 2011). As a result, RCL plays a critical role in the efficiency and the specificity of serpin inhibition (Huntington, Read & Carrell, 2000; Gettins, 2002). Such mechanism of action was reported for prokaryotic and eukaryotic serpins. Interestingly, serpin family is distinguishable by the fact that the native fold is not the most stable form (Gettins, 2002).

### Mechanism of inhibition

Many studies proved that serpins inhibit their targets by an irreversible substrate-like mechanism (Lawrence et al., 1995; Huntington, 2011; Khan et al., 2011). Upon inhibition, both molecules undergo extreme conformational changes that generate a stable covalent serpin-protease complex (Huntington, Read & Carrell, 2000; Khan et al., 2011). Initially, catalytic serine/cysteine of serine/cysteine peptidases performs a nucleophilic attack on the RCL within the scissile bond P1-P1′. Such hydrolysis reaction generates the cleavage of the peptide bond P1-P1′ and the formation of a covalent acyl-ester linkage between P1 and the catalytic serine (Fig. 1B). Then, the RCL is inserted between the A *β*-sheets allowing the translocation of the protease on the opposite side of the serpin. Such structural changes strongly distort the protease active site and both proteins are inactivated by this suicide inhibition mechanism (Lawrence et al., 1995; Wilczynska et al., 1995; Huntington, Read & Carrell, 2000) (Fig. 1A). Several studies highlighted serpin structure-function relationships based on mutagenesis and molecular engineering strategies (Seo et al., 2000; Im, Ryu & Yu, 2004). It was demonstrated that the serpin native form is a metastable conformation, which is converted to a more stable state during protease inhibition (Kaslik et al., 1997; Im, Ahn & Yu, 2000). Notably, the inhibition efficiency is modulated by the protein flexibility and mainly the RCL (Huntington et al., 1997; Lee et al., 1998; Zhou, Carrell & Huntington, 2001). Indeed, it was demonstrated that numerous mutations in the RCL increased the protein stability and significantly reduced the inhibition efficiency (Im, Seo & Yu, 1999; Im & Yu, 2000; Seo et al., 2000; Im, Ryu & Yu, 2004; Jung, Na & Im, 2004).

### Bacterial serpins

The presence of serpins was believed to be restricted to eukaryotes and virus (Irving et al., 2002; Silverman et al., 2010). Owing to recent advances in sequencing technology and the development of bioinformatic tools, new additional serpins were identified in bacteria, protozoa and fungi. Serpins constitute the most distributed superfamily of protease inhibitors across all major branches of life (Irving et al., 2002; Gettins, 2002; Silverman et al., 2010; Harish & Uppuluri, 2018). Studies on bacterial serpins provided limited data regarding their origin and potential functions. The presence of genes encoding serpins in all life kingdoms suggests that such superfamily firstly appeared in prokaryotes before the divergence of the major domains of life (Irving et al., 2002). The loss of serpin genes by some prokaryotes during evolution can be related to the surrounding environment. However, the sporadic presence of serpins in prokaryotes did not support such hypothesis (Irving et al., 2002; Kantyka, Rawlings & Potempa, 2010). The second hypothesis proposes that serpin-encoding genes appeared first in eukaryotes and were acquired by prokaryotes through horizontal gene transfer (Irving et al., 2002). Such statement is challenged by serpins having a competing microbes and modulating the host immune response including that from gingival crevice (Eckert et al., 2018). Several reports supporting the latter hypothesis were described (Irving et al., 2002; Roberts et al., 2004; Goulas et al., 2017). However, as far as we know no evidence exists to reinforce one hypothesis over another.

### Phylogenetic study

**Figure 2:**
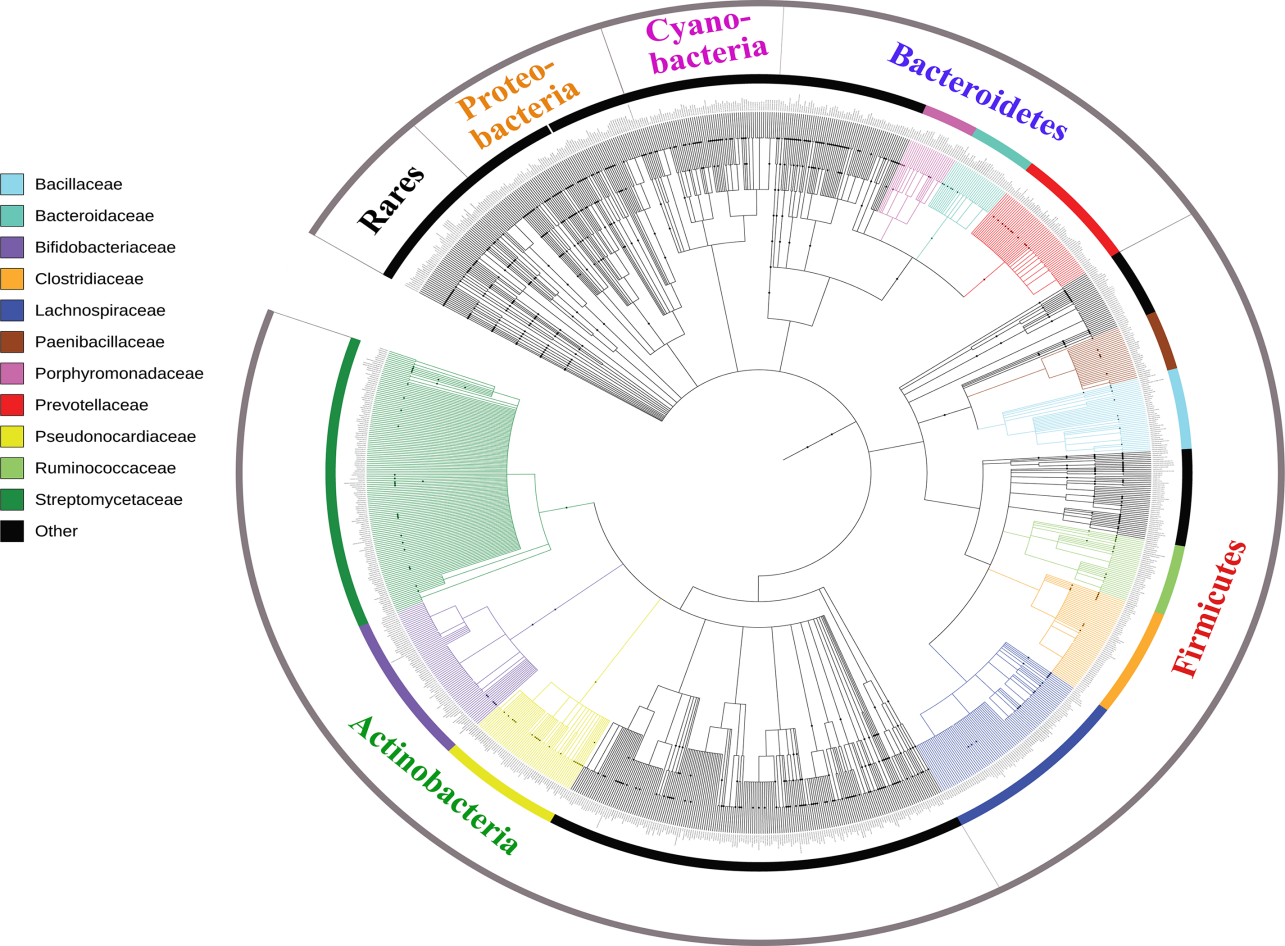
Bacterial serpins distribution. Protein sequences and their taxonomic assignation were retrieved from public database NCBI. Taxonomic lineages are represented in different colors. Phylogenetic tree was built with PhyloT (https://phylot.biobyte.de/) and ITOL.

Analysis of serpins available in the public databases (NCBI) demonstrated that these bacterial antiproteases are distributed in different phyla, mainly Actinobacteria, Firmicutes, Bacteroidetes, Cyanobacteria and Proteobacteria (Fig. 2). In order to explore the distribution of these serpins within each phylum, we carried out a phylogenetic study at the family level (Fig. 2). We noted a significant proportion of serpins that were only represented in a small number of species (<50 species) of a given family which we classified as rare.

In addition to rare families, we found that serpins from the Actinobacteria phylum were mainly distributed in three families: Streptomycetaceae, Bifidobacteriaceae and Pseudonocardiaceae. In the Bacteroidetes phylum beside rare families, serpins belong to the Prevotellaceae, Bacteroidaceae and Porphyromonadaceae families. In Firmicutes, serpins were found in five families: Lachnospiraceae, Clostridiaceae, Ruminococcaceae, Bacilliaceae and Paenibacillaceae while in Proteobacteria and Cyanobacteria, serpins are only found in rare families (Fig. 2). However, in the other phyla there is less diversity at family level but with more abundant bacteria encoding for serpins. We propose that the high abundance of serpins in a given bacterial family could be linked to the adaptation of these bacterial groups to their environments.

### Ecological niches

**Figure 3:**
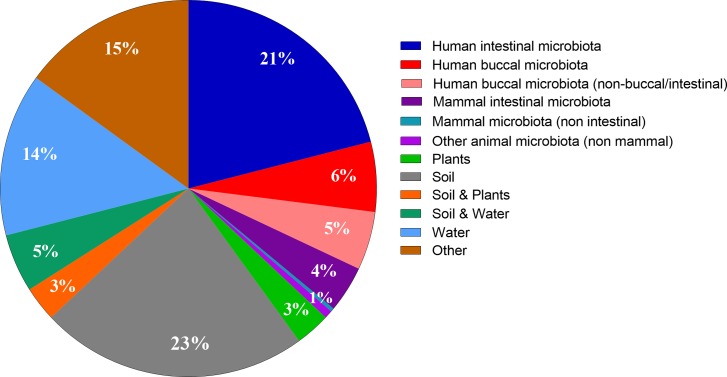
Distribution of bacterial serpin in ecological niches. The pie-chart represents the relative percentage of serpins in various ecological niches.

According to current knowledge, the main ecological niches housing bacteria harboring serpins are: human microbiota (32%), soil (23%) and water (14%) (Fig. 3). These results confirm the finding of Kantyka, Rawlings & Potempa (2010) who reported that serpins belong mainly to benign environments (Kantyka, Rawlings & Potempa, 2010).

Taking into account the wide distribution of serpins in prokaryotes and the lack of data about their regulation and role, the physiological functions of these protease inhibitors remain elusive. Nevertheless, the variability of the ecological niches of the bacterial species encoding serpins stressed that these inhibitors have evolved to perform key functions.

### Thermophilic bacterial serpins

Prokaryotic serpins were initially observed in archaea and some extremophilic bacterial genera (Irving et al., 2002). Sequence analysis of serpins from thermophilic bacteria predicted that these proteins were protease inhibitors (Irving et al., 2002). Indeed, thermopin, a serpin produced by the thermophilic bacterium *Thermobifida fusca*, was first studied and shown to inhibit chymotrypsin. Such inhibitory function was further confirmed by the formation of a covalent complex with the target protease (Irving et al., 2003). Thermopin was also shown to be stable at 60 °C, at which the *α*-1-antitrypsin rapidly lost its activity (Irving et al., 2003). Structural analyses revealed that thermopin exhibits a C-terminal extension (amino acid: 363-367) interacting with Glu309 and Arg258 residues in the s5A and s6A *β*-strands respectively. This takes more importance if we consider that Glu309 and Arg258 residues are highly conserved among serpins and particularly important for the stability of these proteins (Irving et al., 2003).

The serpin from the extremophilic bacterium *Thermoanaerobacter tengcondensis* was further characterized. This serpin, tengpin, inhibits the human neutrophil elastase and forms a covalent complex typical of inhibitory serpins. Like thermopin, tengpin is distinguishable by a structural feature allowing to operate at extreme temperatures (Zhang et al., 2007). In fact, mutagenesis and X-ray studies demonstrated that this serpin displays an N-terminal extension that is essential to stabilize the native metastable status of tengpin (Zhang et al., 2007).

To better investigate the role of serpins in bacteria, three additional serpins were also characterized from the thermophilic bacterium *Clostridium thermocellum* (Kang et al., 2006). This strain has a high ability to degrade cellulose using a multi-enzyme complex, the cellulosome, and exhibits three distinct serpins. Clotm-serpin 1 and Clotm-serpin 2 were predicted as cellulosomal proteins while Clotm-3 is a membrane protein. Biochemical characterization revealed that Clotm-serpin 1 inhibits the bacterial subtilisin. As *C. thermocellum* displays a subtilisin-encoding gene, it was suggested that its serpins are specific inhibitors of bacterial proteases, including its own subtilisin-like protease (Kang et al., 2006). Taking into account these data, bacterial serpins were proposed to protect the cellulosome structure through the regulation of endogenous and exogenous proteases (Kang et al., 2006; Cuív et al., 2013).

### Serpins from the human microbiota

To date, only few serpins from the human microbiota were studied (Ivanov et al., 2006; Ksiazek et al., 2015; Mkaouar et al., 2016; Goulas et al., 2017). A novel serpin from *Tanerella forsythia*, miropin, was characterized and shown to display a broad range of inhibition including serine and cysteine proteases such as neutrophil elastase, cathepsin G, trypsin, and papain (Ksiazek et al., 2015; Goulas et al., 2017). Besides host proteases, miropin inhibits bacterial protease like gingipain and subtilisin (Ksiazek et al., 2015; Goulas et al., 2017). Therefore, it was suggested to act as a virulence factor protecting the bacterium from host and endogenous proteases (Ksiazek et al., 2015). Three serpins from the human gut microbiota were also studied. In fact, the Bifidobacteria genome sequencing revealed the presence of a serpin-encoding gene (Schell et al., 2002; Turroni et al., 2010). Based on transcriptomic studies using Bifidobacterium strain, Turroni et al. (2010) reported the up-regulation of various genes including serpin in presence of proteases (Turroni et al., 2010). Recently, a serpin from *B. longum* has been characterized and reported to inhibit the human neutrophil elastase (Ivanov et al., 2006). A stable covalent complex serpin-protease was further observed when incubating purified serpin with fecal proteases from mice (Ivanov et al., 2006). This serpin was recently reported to prevent enteric neurons activation by supernatants from irritable bowel syndrome patients (Buhner et al., 2018). Such data stressed the potential key role of bacterial serpins to improve gastrointestinal symptoms. Lately, we reported the biochemical characterization of two putative serpins from the human gut bacterium *Eubacterium sireaum* and supposed to be secreted in the intestinal lumen (Mkaouar et al., 2016). The analysis of these novel bacterial serpins, called Siropins, revealed that they efficiently inhibit the human neutrophil elastase and proteinase 3. Interestingly, Siropins are the first bacterial serpins that significantly inhibit the human proteinase 3, known to be involved in IBD. Kinetic studies demonstrated that Siropins were highly efficient in comparison to other bacterial serpins including that of *B. longum*. Furthermore, siropins exhibit a high efficiency to inhibit fecal proteases issued from mice with chemically induced colitis (Mkaouar et al., 2016). This highlights the importance of serpins from the human gut microbiota to inhibit proteases related with human physiopathologies.

## Conclusions

In this review, we analyzed human serpins and their functions to maintain homeostasis as well as their involvement in several diseases. Such data stressed the key role of human antiproteases and highlighted their potential to establish innovative therapeutic strategies. In contrast, bacterial serpins remain today poorly studied. The emergence of metagenomics allowed the identification of new bacterial serpins. Phylogenetic study of this protein family demonstrated that bacterial serpins essentially belong to five phyla colonizing benign environments. The distribution of the serpins in ecological niches showed that the human gastrointestinal tract harbors an elevated number of serpins. The relevance of these bacterial proteins was reinforced through (i) the determination of their efficiency to inhibit fecal proteases recovered from mice with chemically induced inflammation and (ii) the inhibition of human proteases involved in IBD. Above all, it will be interesting to characterize more microbial serpins and to further explore their therapeutic potential. Resolution of the structure of serpin-protease complexes will bring useful structural insights to investigate the serpins structure-function relationships that will allow the improvement of their efficiency and specificity through engineering approaches. Such analysis will promote the use of bacterial serpin mainly in biomedical applications including the set out of new therapeutic alternatives against protease-related diseases.

## Additional Information and Declarations

### Data Availability

The following information was supplied regarding data availability: This is a literature review and does not have raw data.
